# Insights into vaccines for elderly individuals: from the impacts of immunosenescence to delivery strategies

**DOI:** 10.1038/s41541-024-00874-4

**Published:** 2024-04-10

**Authors:** Yingying Hou, Min Chen, Yuan Bian, Yuan Hu, Junlan Chuan, Lei Zhong, Yuxuan Zhu, Rongsheng Tong

**Affiliations:** 1 Department of Pharmacy, Sichuan Academy of Medical Sciences & Sichuan Provincial People’s Hospital, School of Medicine, University of Electronic Science and Technology of China, Chengdu, 610072 China; 2 Personalized Drug Therapy Key Laboratory of Sichuan Province, School of Medicine, University of Electronic Science and Technology of China, Chengdu, 610072 China

**Keywords:** Adjuvants, Pharmaceutics

## Abstract

Immunosenescence increases the risk and severity of diseases in elderly individuals and leads to impaired vaccine-induced immunity. With aging of the global population and the emerging risk of epidemics, developing adjuvants and vaccines for elderly individuals to improve their immune protection is pivotal for healthy aging worldwide. Deepening our understanding of the role of immunosenescence in vaccine efficacy could accelerate research focused on optimizing vaccine delivery for elderly individuals. In this review, we analyzed the characteristics of immunosenescence at the cellular and molecular levels. Strategies to improve vaccination potency in elderly individuals are summarized, including increasing the antigen dose, preparing multivalent antigen vaccines, adding appropriate adjuvants, inhibiting chronic inflammation, and inhibiting immunosenescence. We hope that this review can provide a review of new findings with regards to the impacts of immunosenescence on vaccine-mediated protection and inspire the development of individualized vaccines for elderly individuals.

## Introduction

Aging is a common, inevitable, and complex process in nature^1^, and the aging population is a worldwide concern. Currently, the proportion of elderly individuals is rapidly increasing worldwide. By 2050, the global population over 60 years old will increase to 2.1 billion^2^. Aging is accompanied by a decline in physiological function and progressive immune system degradation, which increases the risk and severity of infectious diseases, malignant tumors, and various chronic diseases. In particular, patients who died from the novel coronavirus disease 2019 (COVID-19) infection were mainly older individuals suffering from underlying conditions^3^, such as obesity, heart disease, cancer, hypertension or high blood pressure, lung disease, and diabetes. Therefore, the public health service and healthcare systems are facing severe challenges caused by aging.

Vaccination is one of the most important successes of modern medicine and is a powerful weapon for preventing and treating fatal infectious diseases^4^. Over the years, vaccines targeting multiple pathogens have saved hundreds of millions of lives^5,6^. People are increasingly aware of the great potential of vaccines in controlling disease outbreaks and protecting older people. Individuals over 65 years are recommended to receive vaccines to prevent severe diseases, such as influenza, herpes zoster, pneumococcal disease, respiratory syncytial virus infection, tetanus, and diphtheria^7^. Nevertheless, the vaccination efficiency in elderly individuals is often reduced due to immunosenescence^8^. For example, the capacity of influenza vaccines to induce immune protection is age-related, with efficacy ranging from 70% to 90% in young people but decreasing to 30–50% in people over 65 years^9^.

The concept of immunosenescence was first proposed by ref. ^10^. Subsequently, scientists began to deeply study the mechanism and role of immunosenescence to find key biomarkers that could guide the prevention and treatment of aging-related diseases. Immunosenescence is a complex process that involves organ reorganization and numerous regulatory processes at the cellular level. The decline in the amount and function of immune cells results in impaired immune responses. Therefore, elderly individuals are at the greatest risk of infection and the most challenging to protect by vaccination^11^. With the aging of the global population and the increased risk of epidemics, the research and development of adjuvants and vaccine systems that offer high-efficacy immune protection for elderly individuals is key for healthy global aging and severe disease prevention. Extensive knowledge of immunosenescence, its impact on vaccination, and its immunological mechanisms is needed to provide scientific insights to optimize vaccines for elderly individuals.

In this review, we analyzed alterations mediated by immunosenescence at the cellular and molecular levels and reviewed emerging strategies to boost the potency of vaccines in elderly individuals, including increasing the antigen dose, preparing multivalent antigen vaccines, adding appropriate adjuvants, inhibiting chronic inflammation, and inhibiting immunosenescence. The prospects and challenges of the various design strategies are also discussed. We hope to incite deep interest in individualized vaccines and provide innovative thoughts to improve vaccines for elderly individuals.

## Impact of immunosenescence on vaccine efficacy

Vaccination capacity is often evaluated based on the level of antigen-specific antibodies in sera, especially antibodies with neutralizing ability, which reflects the immediate responses to pathogens. The memory B cells that are formed through vaccination are also crucial and are quickly activated, expanded, and secrete effector antibodies when confronted with a pathogen reinfection. Meanwhile, in chronic viral infections and tumors, there is an increasing amount of focus on the role of CD4^+^ and CD8^+^ T cell responses^6,12^; these T cells secrete effector molecules that can inhibit pathogens directly or indirectly.

The immunological and molecular signatures of elderly individuals are different from those of young individuals. A study on the comprehensive characteristics of immune responses in the elderly (≥65 years old) showed that those who did not respond to the influenza vaccine presented with multiple states of immune inhibition^13^. In general, the number and function of naïve B and T cells in older individuals are reduced, resulting in weakened immunity to neo-antigens. Studies have shown that aging immune cells can generate a sufficient primary antibody response, although at a slower rate and with a lower ability to neutralize pathogens^14^. Meanwhile, the CD4^+^ and CD8^+^ T cell responses are also diminished^15^.

**Fig. 1:**
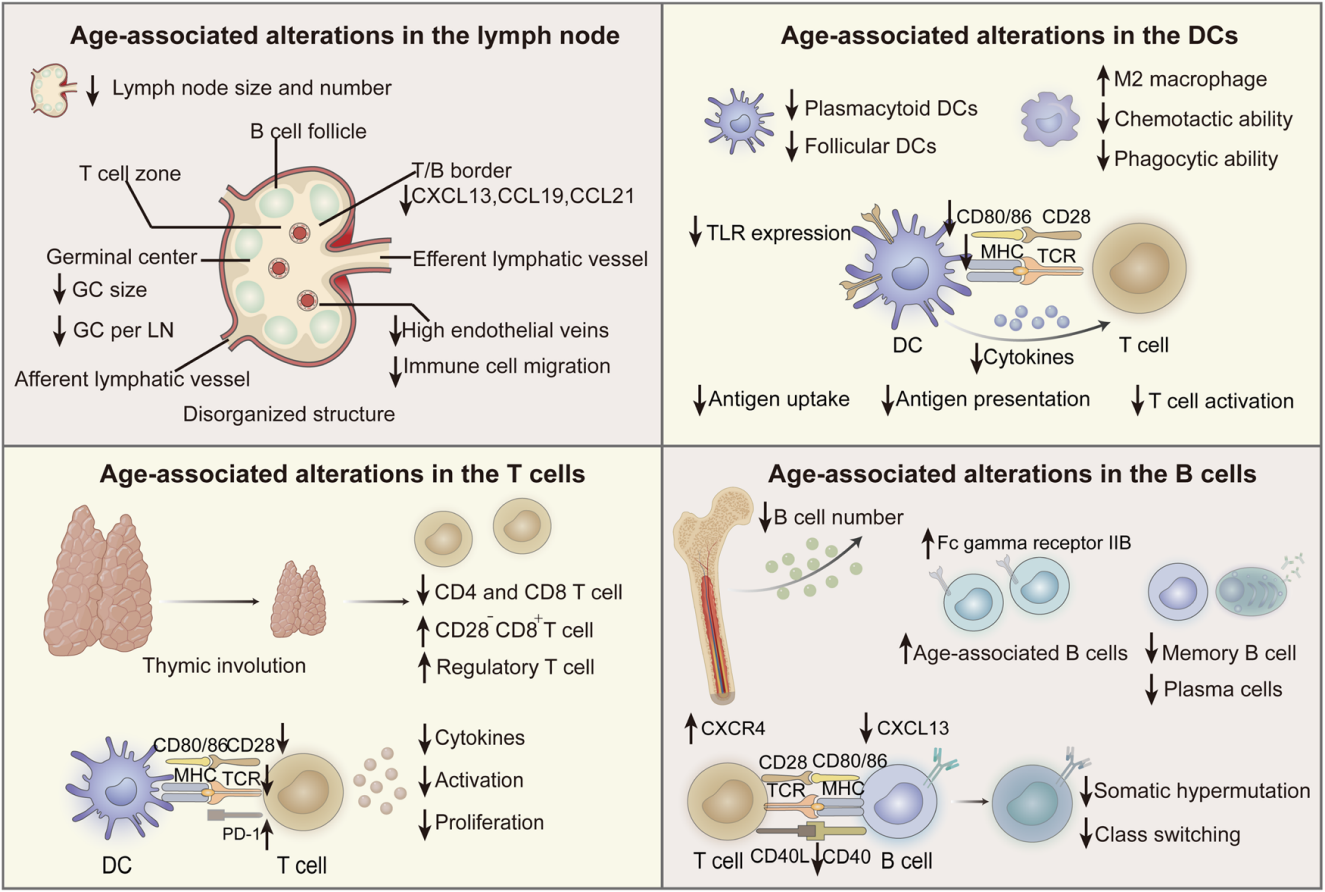
Age-associated alterations in the lymph nodes, DCs, B cells, and T cells. GC germinal center, LN lymph node, DC dendritic cell, MHC major histocompatibility complex, TCR T cell receptor, TLR toll-like receptor, PD-1 programmed death receptor-1. (Created with Adobe illustrator and BioRender.com.).

Our group has previously proposed that the spatial delivery of subunit vaccines should undergo an in vivo cascade that involves the following six steps: lymph node targeted delivery, dendritic cell (DC) subset targeting, B cell modulation, antigen uptake, antigen cross-presentation, and antigen-presenting cell (APC) regulation to stimulate T cells^6^. All of these immune steps are impacted during the aging process, thereby altering immunity. The age-associated alterations in lymph nodes, DCs, B cells, and T cells are summarized in Fig. 1.

The lymph node contains plentiful immune cells and is the main location where APCs present antigens to activate T cells, leading to the development of adaptive immune responses^16^. Targeting lymph nodes will induce faster and more direct interactions between vaccines and immune cells, which can lead to effective immune activation. Both the size and number of lymph nodes are reduced with aging. The crucial site of the humoral immune response in lymph nodes is the germinal center (GC). However, the GC area and the intensity of the GC response decrease with age^17,18^. At the same time, the decrease in endothelial veins in lymph nodes and the reduced phosphoinositide 3-kinase and CC-chemokine receptor 7 signals on immune cells limit the migration of immune cells to lymph nodes^19,20^. This change may ultimately lead to weakened lymph node targeting ability.

DCs are critical APCs that initiate naïve T cells, which play a crucial role in bridging innate and adaptive immunity^21^. DCs are divided into several important subgroups: conventional DCs mainly serve to prime naïve T cells; plasmacytoid DCs (pDCs) can produce large amounts of type I interferon (IFN) to participate in antiviral immunity; monocyte-derived dendritic cells (moDCs) can present antigens to effector T cells and secrete cytokines; Langerhans cells (LCs) are epidermal DCs that can present antigens to induce T cell immunity^22^. In addition, follicular DCs are specialized subsets in the lymphoid follicles that promote B cell antigen presentation^23^. The frequencies of circulating pDCs and follicular DCs decrease, while the number of circulating moDCs remains unchanged with age^8^. After being internalized by APCs, the antigen is processed into a peptide. Then, the antigen peptide and major histocompatibility complex (MHC) molecules generate the first signal presented to T cells. During this process, APCs gradually mature to express costimulatory molecules (the second signal) and secrete cytokines (the third signal). These cytokines, such as interleukin (IL)-12, IL-4, and IL-6, assist in the polarization of T cells into Th1, Th2, or Th17 cells. As age increases, the overall number of lymphocytes decreases and transfers toward myeloid cells. As such, there is a decrease in the number of DCs with increasing age^24^. The expression of toll-like receptor (TLR) 3, 7/8, costimulatory molecules, and MHC molecules on DCs decreases with age^8^. After stimulation with TLR7 or TLR9, the pDCs of elderly individuals also show reduced secretion of tumor necrosis factor (TNF) -α and IFN-α^25^. Therefore, the phagocytosis, antigen presentation, and cytokine secretion abilities of DCs decrease, which further hinders subsequent T cell activation and immune responses. Furthermore, distinct vaccines have been shown to stimulate different subsets of DCs^26^. Identifying the diverse impacts of immunosenescence on DC subsets is pivotal for developing vaccines for older individuals. Macrophages serve as key APCs; with age, macrophages have impaired chemotactic and phagocytic abilities. In older mice, a high frequency of M2 macrophages was observed in the spleen and lymph nodes, which have abilities of angiogenesis and tumor growth^27^. The researchers proposed that macrophages from older mice are particularly sensitive to signals that promote their development into highly immunosuppressive M2 macrophages, which promote tumor development. IL-2/anti-CD40 antibody immunotherapy to activate macrophages can rescue age-related T cell dysfunction. Similarly, Prieto et al. found that senescent alveolar macrophages counteract cytotoxic T-cell accumulation to promote lung tumorigenesis^28^. Surprisingly, therapeutic interventions targeting senescent macrophages by knocking out their specific expression molecules can delay tumor progression. These observations may inspire the design of cancer vaccines for elderly individuals. M2 macrophages can be targeted for removal by specific expression molecules, or their polarization and immunosuppression can be corrected to improve the efficacy of vaccines in the elderly.

B cells are the center of humoral immunity and can be activated, amplified, and secrete protective antibodies that respond to immunization. At the same time, B cells are also APCs that can present antigens following their recognition via surface of B cell receptors^29^. The bone marrow is the main source of B cells. With increasing age, the hematopoietic function of the bone marrow decreases, which leads to a decline in the number of B cells. Studies have shown that the number of naïve B cells decreases with age, while memory B cells generally remain stable^30^. However, Pritz et al. found that the frequency of naïve B cells remains unchanged or increases with age, and the number of plasma cells and memory B cells decreases^8,31^. This discrepancy could be due to the use of different markers to define B cell subgroups. Additionally, differences in the age groups and cohort sizes of the analyzed samples may also affect the study results. At the same time, the age-associated B cells (ABCs) in both mice and humans are antigen-experienced memory B cells generated during microbial infections and vaccinations^32^. Aging mice showed an increased number of ABCs, which secrete cytokines to impair the generation of young pro-B cells^18^. Yam-Puc et al. reported that increased ABC frequency in humans is associated with reduced antigen-specific memory B cells and decreased neutralizing capacity against SARS-CoV-2^33^. They also identified that increased expression of the inhibitory receptor Fc gamma receptor IIB can bind immune complexes to enhance their clearance, contributing to reduced vaccine response.

The GC is polarized into two functionally distinct regions: the light and dark zones. In the light zone, the B cells capture antigens and present them to the T follicular helper (Tfh) cells. In the dark region, the B cells proliferate and mutate, improving the specificity and affinity of the antibodies. Tfh cells through the CXCR5/CXCL13 recognition position to the light zone of the GC and through the CXCR4/CXCL12 recognition position to the dark zone of the GC^34^. CXCL13 is a chemokine in B cell follicles that is pivotal for transporting Tfh CD4 cells to B cell follicles during the humoral immune response^35^. The level of CXCL13 also decreases with age^36^. Immunosenescence reduces naïve CD4 T cell differentiation into Tfh cells, so there is an insufficient amount of T cells to assist the B cell response^37^. Silva-Cayetano et al. found that the expression of CXCR4 increased in Tfh cells from aged mice, while the expression of CXCR5 did not change^38^. The increased expression of CXCR4 led to enhanced chemotaxis of CXCL12 in the dark zone of GC, directly decreased the amounts of antigens presented to B cells, and decreased specific antibody responses. Moreover, research has shown that the expression of activation-induced cytidine deaminase necessary for affinity maturation and class switching in B cells is significantly reduced in elderly individuals^39^, which subsequently leads to decreased affinity and antibody responses. However, Stiasny et al. found that vaccination against tick-borne encephalitis can induce high avidity of antibodies and functional activity even in the elderly^40^. These alterations in B cells may explain the impairment in protective humoral immunity in older individuals after vaccination.

As the key effector cells of immune responses, T cells are also impacted by aging more than other cells. The replenishment of T cell frequency is achieved through export by the thymus and the self-renewal of peripheral naïve T cells^41^. There is a loss of naïve T cells in elderly individuals caused by thymic involution. Moreover, the expression of the T cell receptor (TCR) and costimulatory molecules CD27 and CD28 decreases, which inhibits T cell stimulation and proliferation in elderly individuals following vaccination^8^. The lack of CD27 and CD28 expression in older individuals is a feature of T cell senescence, presumably caused by repeated T cell activation. The mechanism underlying the reduced expression of CD27 and CD28 deserves further research; this information could be used to design methods to restore T cell function and improve immunity in elderly individuals. Meanwhile, the expression of programmed death receptor-1 (PD-1) on T cells increases with age, while the amount of CD28^-^CD8^+^ T cells and regulatory T (Treg) cells that inhibit T cell activation and proliferation increases^7,8^. In particular, CD28^-^CD8^+^T cells inhibit T cell activation and expansion by producing TGF-β, IL-7, and IL-10^42^. T cells with high expression of PD-1 inhibit T cell activity through the PD-1/programmed death ligand 1 (PD-L1) pathway, thereby inhibiting the ability of T cells to kill tumor cells in the tumor microenvironment and allowing the immune escape of tumors^43^. Thus, these changes in senescent T cells play an essential role in the reduced immune response following vaccination. The deficiency of CD4^+^T cells in older individuals also markedly affects immunity. Tfh cells are a subgroup of CD4^+^T cells, which helps antigen-specific B cell proliferation, class switching, and somatic hypermutation^44^. Compared with young individuals, the number of circulating Tfh (cTfh) cells is markedly reduced in elderly individuals. In addition, coculture of cTfh cells from older individuals with allogeneic naïve B cells from a young subject led to impaired expansion and reduced antibody production^45^. Therefore, strategies to enhance specific Tfh cell stimulation could improve vaccine-induced immunity.

## Inflammation and immunity

Immunosenescence is accompanied by the development of a chronic and systemic sterile inflammatory environment called inflammaging^46^. The characteristic factors in this environment include C-reactive protein, TNF-α, IL-6, and other proinflammatory cytokines; the levels of these factors are higher in older adults than in young people^47^. The acute inflammatory reaction is essential in triggering immunity against invasive pathogens. After initial inflammation, the amount of inflammation is reduced for a period to avoid continuous tissue damage and restore tissue homeostasis^48^. Nevertheless, research has shown that chronic inflammation negatively impacts immunity because increases in the inflammatory response reduce the effectiveness of vaccines^49^.

Chronic inflammation during aging is caused by multiple mechanisms. Chronic viral infections, such as cytomegalovirus, can induce lifelong latent infections after the initial infection, periodic reactivation of the virus, and the initiation of subclinical immunity^50^. During aging, the proportion of muscle decreases and visceral fat increases. Obesity, especially visceral fat accumulation, can induce inflammatory sites that infiltrate monocytes, phagocytes, B cells, and T cells, which produce inflammatory cytokines^51^. Gut permeability increases with age, which causes the leakage of intestinal contents into the bloodstream. For example, lipopolysaccharides in circulation can trigger inflammatory cytokine secretion by monocytes via pattern recognition receptor (PRR) activation^52^. Moreover, changes in the microbiome in elderly individuals may also elevate the level of circulating inflammatory cytokines^53^.

Damage-associated molecular patterns are cellular products that are released during stress, injury, or death and include calcium-binding proteins, histones, ATP, uric acid, and heparin sulfate^49^. When faced with acute inflammation, there is a decrease in efferocytosis and the clearance of apoptotic neutrophils during the regression phase of inflammation. Therefore, the ability to resolve inflammation in elderly individuals is weakened compared to that of young individuals^54^. Meanwhile, senescent cells secrete multiple inflammatory cytokines (such as IL-1β, IL-6, and TNF-α), chemokines (such as CCL2 and IL-8), and growth factors. This secretion of proinflammatory mediators is called the senescence-associated secretory phenotype (SASP), which contributes to the inflammatory microenvironment^55^. In addition, the signaling pathway of cyclic GMP-AMP synthase and stimulator of interferon genes (STING) plays a key role in the development of chronic inflammation and functional decline during aging^56^. The NOD-, LRR- and pyrin domain-containing protein 3 (NLRP3) inflammasomes, which promote the maturation and release of IL⁃1β and IL⁃18, are involved in a variety of host immune and inflammatory responses. Overactivation of the NLRP3 inflammasome can lead to pathological inflammation and is associated with age-related diseases as it is involved in the inflammatory aging process^57^. The nuclear factor kappa-light-chain-enhancer of activated B cells (NF-κB) is a family of transcription factors that also play an essential role in mediating immune and inflammatory responses^8^.

Inflammatory cytokines can increase the expression of inhibitory ligands on immune cells to alter immunity. For example, TNF-α facilitates the expression of PD-L1 on APCs. PD-L1 binds to PD-1 expressed in T cells^58^; and the significantly increased level of PD-1 on senescent T cells inhibits the immune response^59^. Inflammation-related cytokines may also increase the frequency and promote the function of Foxp3^+^ Treg cells in elderly individuals; these cells are recruited to inflammatory sites and inhibit antigen-specific immune responses^60^. In addition, SASP components impact the immune response in various ways. Monocyte-derived and plaque-infiltrating macrophages in elderly patients with coronary artery disease express PD-L1 with high surface density, resulting in suppressed T cell immunity^61^. Prostaglandin E2 can impair the cytotoxic T lymphocyte (CTL) response and suppress CTL survival^62^.

## Molecular hallmarks of aging and immunity

**Fig. 2:**
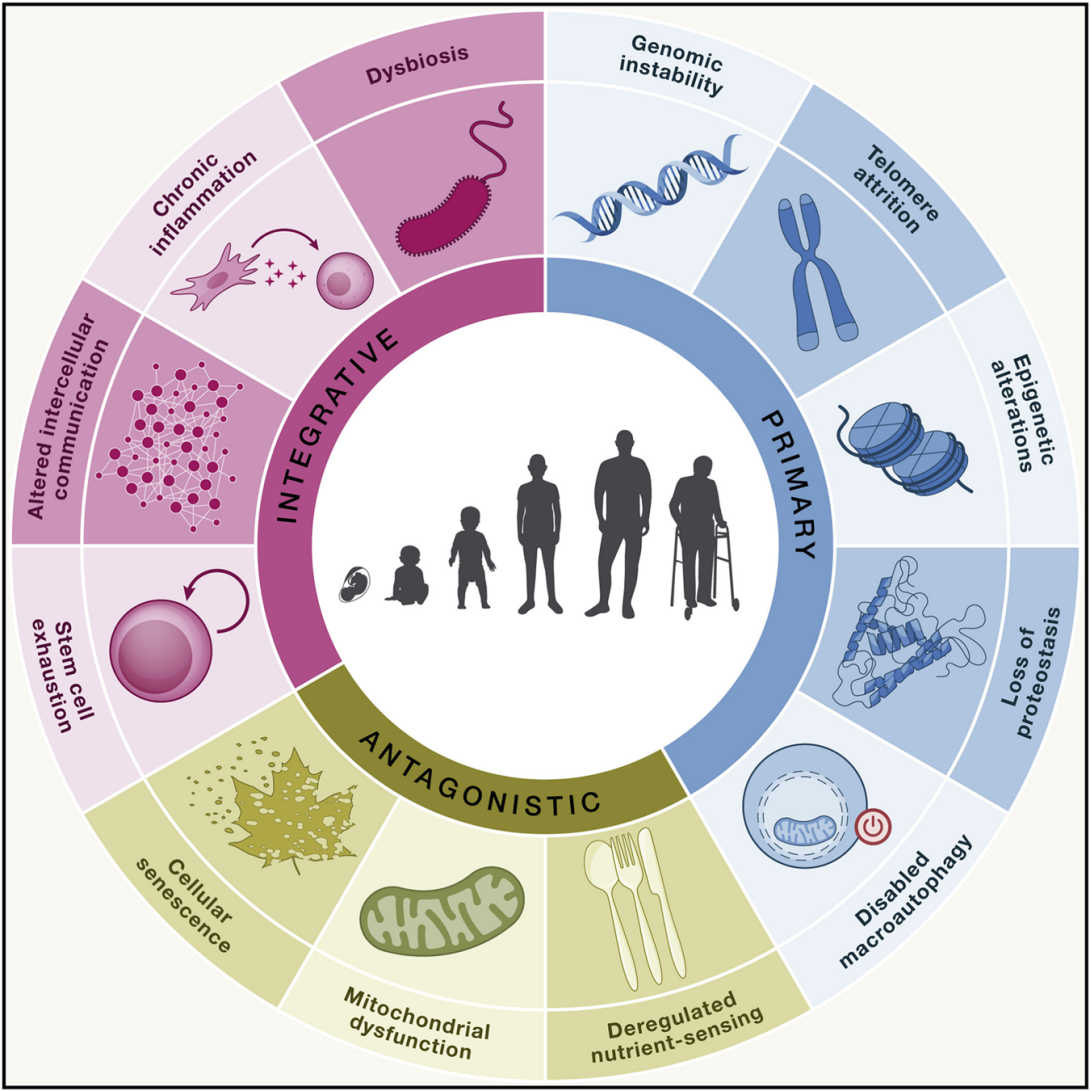
The main hallmarks of aging include genomic instability, telomere attrition, epigenetic alterations, loss of proteostasis, disabled macroautophagy, deregulated nutrient sensing, mitochondrial dysfunction, cellular senescence, stem cell exhaustion, altered intercellular communication, chronic inflammation, and dysbiosis^63^. Copyright 2023, Cell Press.

Recently, important advances have been made in research on signaling pathways and molecular mechanisms that influence senescence. Researchers have proposed certain molecular hallmarks of aging (Fig. 2), such as genomic instability, telomere attrition, epigenetic alterations, loss of proteostasis, compromised autophagy, and mitochondrial dysfunction^63,64^. Considering our background knowledge of vaccine-induced immune responses, we speculate that compromised autophagy, the loss of proteostasis, and telomere attrition may affect the immune responses induced by vaccines.

Autophagy activity decreased in different tissues of different species with age. Autophagy gene transcripts, such as ATG5, ATG-7, and BECN1, decreased with age^65^. Recent findings support the idea that the reduction in autophagy is the core molecular mechanism underlying immunosenescence^66^. Autophagy is a highly conserved process that can degrade defective intracellular organelles and misfolded protein aggregates^67^. Autophagy can prolong life by inhibiting the mammalian target of rapamycin (mTOR) signaling pathway or activating the adenosine 5′-monophosphate-activated protein kinase (AMPK) pathway^68^. In aging, damaged proteins and organelles gradually accumulate. Cell quality control systems are dysfunctional, such as the unfolded protein response and autophagy, resulting in the reduced antigen presentation by APCs^69^. Wang et al. conjugated poly (β-amino ester) with an autophagy-inducing peptide to self-assemble a nanovaccine^70^. The results showed that improving antigen cross-presentation and the vaccine-induced immune response are promoted by autophagy in DCs. Autophagy can lead to the formation of antigen storage compartments in APCs, prolong the allowed storage time of antigens, and facilitate antigen presentation and subsequent initiation of CD8^+^ T cell reponses^71^. In addition, autophagy is pivotal in regulating T cell responses. The loss of autophagy results in reduced T cell survival, proliferation, and cytokine secretion following TCR activation^72^.

Spermidine is a natural polyamine that can improve the health and extend the lifespan of entire species^73^. Spermidine, as an autophagy agonist^74^, is undergoing clinical trials to investigate its ability to restore autophagy in T and B cells to improve vaccine potency in elderly individuals (NCT05421546). Metformin is a drug used to treat type 2 diabetes safely and can be used for children and even pregnant women. Metformin can induce cell autophagy by activating the AMPK signaling pathway^75^, inhibiting the stimulation of the NLRP3 inflammasomes, and improving aging-related inflammatory rresponses^76^. A completed clinical trial verified that metformin can elicit more effective immune responses in elderly individuals given the influenza vaccine (NCT03996538). Another study also investigated whether metformin can improve immune responses to the pneumococcal conjugate vaccine in older individuals (NCT03713801). Future research may focus on utilizing spermidine or metformin in vaccine design to elevate immunity in elderly individuals.

With extensive research on the molecular mechanism of aging, it has been found that telomeres stimulate molecular pathways that drive the aging process and related diseases^77^. Telomeres are a group of repetitive sequences that protect the ends of chromosomes and prolong the cellular lifespan. As the clock of life, telomeres gradually shorten with cell division and aging. Telomerase can prolong telomere repair to alleviate telomere loss caused by T cell proliferation, but telomerase activation is insufficient in protecting T cells from immunosenescence. Recent studies have shown that APCs form immunological synapses with TCRs through MHC and present antigens to T cells. T cells elongate their telomeres by acquiring telomeres in extracellular vesicles (EVs) from APCs^78^. In vivo experiments in mice found that telomere-containing EVs can elicit antigen-specific T cell expansion, stem cell-like memory T cells, and central memory cell proliferation. Additionally, a comparative experiment using the influenza vaccine indicated no significant difference in the short-term protection of the vaccine based on telomere EVs. However, the long-term protection of the telomere EV group was dramatically better than that of the group without telomere EVs. This finding further showed that telomere-containing EVs improved immune memory and long-lasting immunity. The results of this study suggest that vesicles carrying telomeres may be a potential strategy for inhibiting T cell immunosenescence and provide new ideas for the design of vaccines for elderly individuals.

**Fig. 3:**
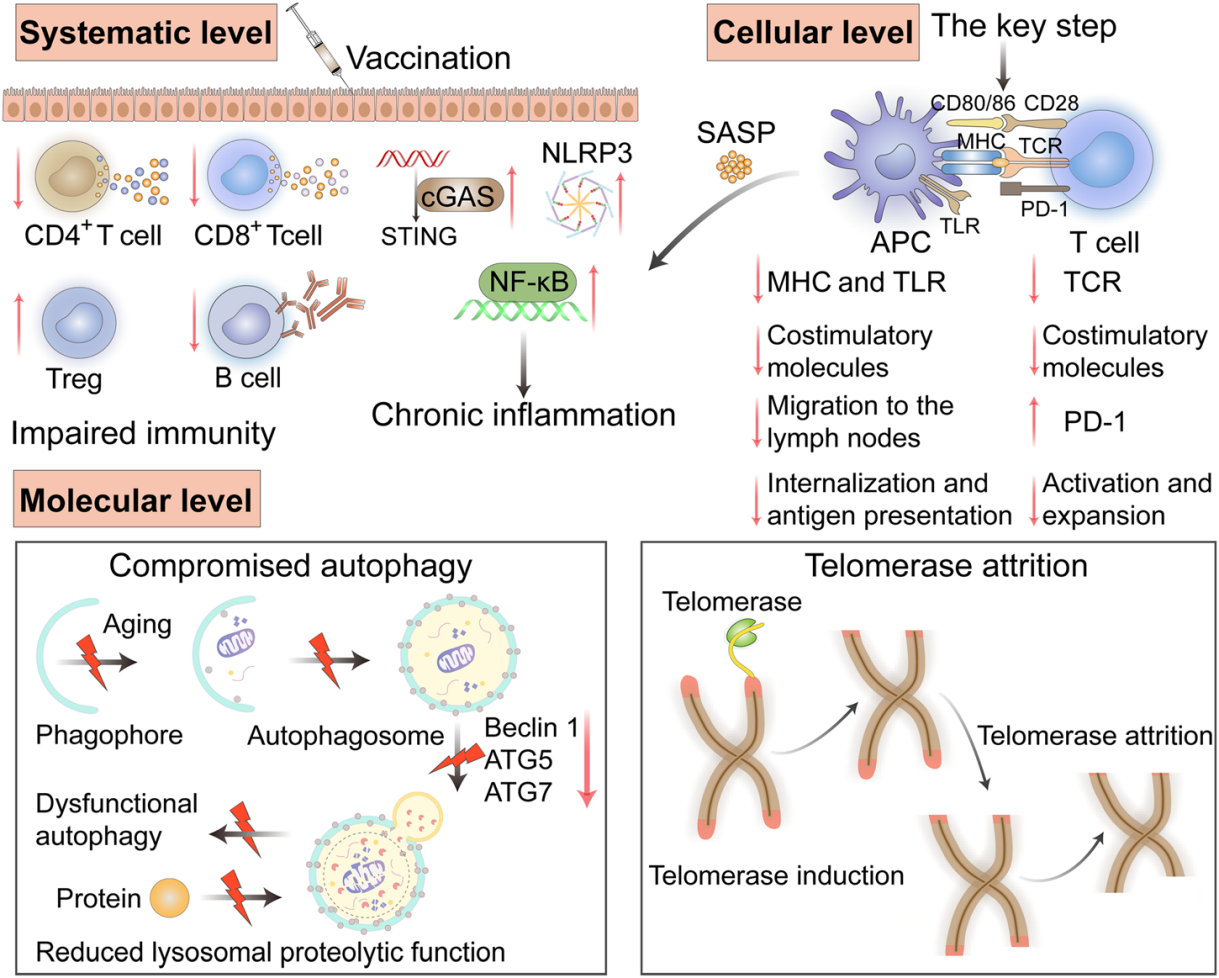
The characteristics of changes in immunosenescence at the systematic, cellular, and molecular levels in elderly individuals. The generation and maintenance of protective immunity induced by vaccination are weakened. The cGAS-STING, NLRP3 and NF-κB signaling pathways were upregulated, leading to chronic inflammation. Meanwhile, the expression of MHC, TLRs, and costimulatory molecules on APCs is reduced, leading to reduced migration to the lymph node, internalization, and antigen presentation of APCs. T cells have low expression of TCRs and costimulatory molecules, but high expression of PD-1, which leads to reduced T cell stimulation. At the molecular level, there are mainly compromised autophagy and telomere attrition. STING stimulator of interferon genes, cGAS cyclic GMP-AMP synthase, NLRP3 NOD-, LRR- and pyrin domain-containing protein 3, NF-κB nuclear factor kappa-light-chain-enhancer of activated B cells, SASP senescence-associated secretory phenotype, APC antigen-presenting cell, MHC major histocompatibility complex, TCR T cell receptor, TLR toll-like receptor, PD-1 programmed death receptor-1, ATG5 autophagy-related 5, ATG7 autophagy-related 7. (Created with Adobe illustrator).

We think that the identification of new markers of immunosenescence will inspire vaccine design and development in the future. In conclusion, we summarized the alterations in immunosenescence at the systematic, cellular, and molecular levels in the elderly (Fig. 3).

## Design strategies for vaccines for elderly individuals

Although the vaccine-induced immunity of elderly individuals is significantly reduced, some strategies are still available to enhance protective immunity. Understanding what strategies already exist in this field is imperative for the development of other high-efficacy vaccines for elderly individuals.

### High-dose vaccines

Compared with traditional vaccines, a high-dose influenza vaccine can provide four times the dose of hemagglutinin antigen and improve the immunogenicity and vaccine potency in people ≥65 years old^79^. High-dose influenza vaccine immunization generates a higher number of antibodies and improves protection against influenza virus in elderly individuals^80^. At the same time, the risk of influenza virus infection, hospitalization, and mortality among older people has significantly reduced^81^. We speculate that an increased antigen dose will increase immunogenicity and induce long-lasting T cell and B cell responses. With age, the quantity of naïve T cells and the diversity of TCRs continue to decline^82^, which means that the possibility of naïve T cells meeting the homologous antigen presented by APCs after vaccination is reduced. However, increasing the vaccine dose can help compensate for the decrease in naïve T cells by increasing the density of DCs that present antigens to T cells in secondary lymphoid tissues, which boosts the possibility of productive interactions between T cells and APCs.

From the perspective of delivery carriers, microparticles can encapsulate more antigens than nanoparticles. Studies have reported that particles with large surface areas, such as metal-organic frameworks, mesoporous silica, and Pickering emulsions, have excellent antigen-loading capabilities^83–85^. Moreover, some antigen self-assembled nanoparticles can achieve high-dose antigen delivery without additional carriers^86,87^. These delivery carriers may be useful in high-dose vaccines.

### Multivalent vaccines

An ongoing challenge in vaccination is developing a vaccine that can boost broad protective immunity^88^. Multivalent vaccines are an excellent choice and have proven effective in practice. Multivalent vaccines contain antigens from a variety of strains/serotypes of pathogens, which can produce cross-protection and provide broader coverage of antigenic variable pathogens^89^. Commercially available pneumococcal 13-valent and 23-valent vaccines contain multiple antigen subtypes that can protect against the common serotypes of *Streptococcus pneumoniae* in older adults^90,91^. A comparison of trivalent and monovalent influenza vaccines showed that polyvalent vaccines can evoke broader coverage of heterotypic and cross-protection^92^.

When developing multivalent vaccines, it is first necessary to select a combination of antigens with diverse valence types to ensure their immunogenicity. Subsequently, studies should focus on evaluating whether multivalent vaccines can induce more neutralizing antibodies and cross-neutralization against other viral variants, thereby verifying whether broader protection is achieved.

### Adjuvanted vaccines

Adjuvants are substances that can improve vaccine efficacy through versatile mechanisms, such as promoting proinflammatory cytokine secretion, recruiting and stimulating innate immune cells, activating PRRs expressed on immune cells, and promoting antigen presentation^93,94^. Adding appropriate adjuvants can decrease the required dosage of antigens, reduce the number of vaccinations needed, and broaden immune protection^95^; adjuvant research is an important direction for improving vaccine efficiency. A scoping review showed that several adjuvants such as MF59, AS03, AS01, and CpG oligodeoxynucleotides (CpG-ODN) are effective in older adults^96^. We will present these adjuvants next.

The Fluad® influenza vaccine contains an oil-in-water emulsion MF59 (an oil-in-water emulsion of squalene oil), which was licensed in 1997 in Italy, and is effective in elderly individuals^97^. The addition of MF59 adjuvant reduces the rate of infection and hospitalization of elderly individuals with influenza virus^97^. Studies have shown that the MF59 adjuvant can significantly increase the number of influenza virus antigen-specific antibodies and the serum conversion rate compared to vaccination with no adjuvant^98,99^. MF59 can induce the secretion of cytokines and chemokines, recruit immune cells such as monocytes and neutrophils to the injection site, and promote the transport of antigens to lymph nodes^93^. The oil-in-water emulsion AS03, which was used in another influenza vaccine, also showed high immunogenicity in the elderly^97^. AS03 contains α-tocopherol components, which strongly upregulate the expression of inflammatory cytokines and chemokine coding genes in the lymph nodes^100^. AS03 can also activate the CD4^+^T cell response, thus leading to the continuous production of neutralizing antibodies and more memory B cells^101^. Additionally, the safety and immunogenicity of recombinant influenza vaccines using matrix M-1 saponin complex adjuvants have been studied in older people (NCT03293498). Another adjuvant, AS01, is a liposome-based vaccine adjuvant system that uses saponin QS21 and the TLR4 agonist monophosphoryl lipid A (MPLA)^102^; vaccines using this system showed improved humoral and cellular immunity. MPLA can directly activate APCs through TLR4 activation, thereby stimulating costimulatory molecule expression and cytokine secretion. QS-21 activates caspase-1 in subcapsular sinus macrophages and activates cytotoxic CD8^+^T cells^103^. These two components simultaneously act on the innate immune system to synergistically amplify the immune response. An adjuvanted recombinant herpes zoster vaccine also includes AS01 and glycoprotein E; this vaccine was effective in 97% of people ≥ 50 years old^104^.

Recently, TLR agonists have shown excellent potential in activating the immune response. Considering the low expression of some TLRs in elderly individuals, specific TLR agonists may not be appropriate^105,106^. Reduced expression of TLR3 and TLR8 in human mDC and TLR7 in pDC leads to reduced cytokine secretion after activation; nevertheless, the expression of TLR1, 2, 4, 5, 6, and 9 did not change with age, and decreased cytokine secretion was also observed, suggesting that other unknown factors besides TLR expression influence the immune response^25^. CpG-ODN can activate TLR9 and promote the proliferation and activation of B cells. Compared to the 3-dose schedule of the hepatitis B vaccine adjuvanted with aluminum, the 2-dose schedule of the vaccine adjuvanted with CpG-ODN resulted in higher serum protection levels in older adults^107^. Lim et al. reported that flagellin-dependent TLR5 expression and signaling are well retained in macrophages from older individuals, similar to that seen in macrophages from young individuals^108^. The pneumococcal surface protein A vaccine adjuvanted with flagellin produced a higher level of specific IgG and IgA responses and showed a high protective effect against *Streptococcus pneumoniae* in aged mice. Therefore, flagellin-dependent TLR5 is a promising immune adjuvant for older adults. Denton et al. found that targeting TLR4 via its ligand can increase MAdCAM-1^+^ stromal cell activation and trigger the GC response to immunization^109^. Therefore, age-associated alterations in GC and stromal cell responses can be a target for improving vaccine efficacy in elderly individuals. Other studies also demonstrated that TLR agonist combinations elicited effective immunity in experimental models and may have potential applications in high-efficacy vaccine design for elderly individuals. Zareian et al. reported that the combination of TLR7/TLR8 and TLR4 resulted in higher cytokine secretion by DCs from old adults^110^. This synergistic effect may be due to the combined activation of MyD88 and TRIF-dependent signal transduction pathways.

Useful adjuvants should facilitate innate and adaptive immunity and produce long-lasting protective memory^88,111^. Furthermore, adjuvants must strike a balance between reduced inflammation and the low inflammatory state that may hinder vaccine-induced immunity. In view of the underlying chronic inflammation in elderly individuals, adjuvants should improve APC function without eliciting intense inflammatory responses from APC or other immune cells, such as T cells. Ross et al. illustrated that the novel nanoadjuvants (polyanhydride nanoparticles and pentablock copolymer micelles) and cyclic dinucleotides (a STING agonist) have moderate induction of cytokine secretion and weak inflammatory properties, which may be suitable for older adults^112^. In addition, the impacts of adjuvants on vaccine efficacy often result from various mechanisms. Combinations of adjuvants have attracted widespread attention because they can coordinate their respective advantages to maximally enhance immunity^113^. Nanishi et al. found that the TLR9 agonist CpG-ODN can synergize with aluminum hydroxide adjuvant to promote immune protection generated by the SARS-CoV-2 receptor binding domain vaccine in older mice^114^. Future research should focus on adjuvant combinations to induce more comprehensive immune responses.

### Inhibiting chronic inflammation

There is increasing research showing that preexisting inflammation can determine vaccine reactivity. Therefore, regulating baseline inflammation before vaccination may be a potential idea to elicit immune responses^49,88^. Some studies have attempted to diminish chronic inflammation in older people by administering p38 mitogen-activated protein kinase (MAPK) or mTOR inhibitors before vaccination^115,116^. Local rapamycin treatment before vaccination resulted in a 20% increase in antibody titer and a decrease in the number of inhibitory CD4^+^ and CD8^+^ T cells^117^. Additionally, as the main source of inflammatory factors, removing senescent cells can also be expected to become an approach to enhance the effectiveness of vaccines^118^. Dasatinib, quercetin, fisetin, and navitoclax were explored as anti-senescent cell therapies that act through different mechanisms^119,120^. Moreover, senescent cells upregulated many biomarkers: Senescence-associated β-galactosidase in intracellular lysosomes, which is a classic marker of cellular senescence; CD9 on cell membrane, which regulates many cellular cell adhesion, cell motility, activation, and differentiation; β2-microglobulin, which forms the light chain of MHC-I molecules; and CD47, which functions as a “don’t eat me” signaling molecule^121^. They have shown great potential as targeted delivery ligands for senescent cells.

Metformin is an exciting candidate drug that targets inflammation and inhibits immunosenescence^122^. Metformin was reported to ameliorate Th17 inflammation by inducing autophagy and improving mitochondrial bioenergetics^123^. Other immunomodulators, such as imiquimod and COX-2 inhibitors, were evaluated in terms of their ability to promote vaccine immunity by transiently alleviating chronic inflammation before vaccination^124,125^. However, using immunomodulator drugs to target inflammatory signaling pathways has been successfully tested, but the impacts of long-term inhibition and potential side effects are still unclear. Spermidine has pleiotropic properties, including anti-inflammatory, antioxidant, and molecular chaperone activity; furthermore, spermidine enhances mitochondrial metabolism and modulates protein homeostasis^126^. Many of the anti-aging effects of spermidine are related to its polyamine-induced cytoprotection of autophagy. Spermidine was reported to regulate macrophage differentiation and alleviate colitis symptoms in mice^127^. Epigallocatechin gallate (EGCG) is a natural product extracted from green tea that contains a phenolic structure with three hydroxyl groups with various activities, such as anti-inflammatory, antioxidant, antitumor, and antibacterial activities^128^. Recent studies have found that EGCG can reduce aging-associated NF-κB inflammation and oxidative stress, thereby prolonging the life of healthy rats^129,130^. Interestingly, EGCG also has adjuvant effects when co-administered with influenza virus HA antigen, which induces a large number of neutralizing antibodies^131^. Incorporating these substances with both anti-inflammatory and adjuvant effects into vaccine formulations will be a potential strategy for future vaccine design.

In addition to drugs that target senescent cells and inhibit inflammation, finding target cells that efficiently inhibit inflammation pathways is another strategy. Vaccine-induced ICOS^+^CD38^+^ circulating Tfh cells from elderly individuals stimulate TNF-NF-κB inflammatory pathway activation^132^. Studies suggest that ICOS^+^CD38^+^ circulating Tfh cells are essential for the design of vaccines that target age-related alterations to the inflammation pathway.

### Inhibiting immunosenescence

Currently, the main measures to elevate vaccination efficiency in elderly individuals include increasing the antigen dose, preparing multivalent vaccines, performing multiple immunizations, and adding adjuvants to boost the immune response. These approaches aim to optimize the vaccine delivery system but often ignore the influence of the immune microenvironment of older individuals on the immune response. Without fundamentally improving the immunosenescence of elderly individuals, vaccines will not be able to produce strong and long-lasting immune efficacy. Inhibiting immunosenescence in elderly individuals will be a critical measure to improve immune responses to vaccines. Finding adjuvants and vaccine components that can inhibit immunosenescence is an attractive direction for future research.

An increasing number of studies have shown that T cell aging is the major reason for diseases in elderly individuals^133^. T cells are also vital effector cells for vaccines, exert cellular immunity, and kill infected or tumor cells^134,135^. The ability of T cells to recognize and eliminate pathogenic microorganisms or tumor cells significantly decreases with age^136^. In particular, the immunosenescence of CD8^+^ T cells in tumor-draining lymph nodes enables tumor cells to escape immune surveillance and elimination and promotes tumor progression^137^. Senescent T cells in the tumor microenvironment have become a new target for tumor immunotherapy^138^. However, nanovaccines to reverse senescent T cells have not yet been reported.

Most studies have reported that improving immunity in older people involves inhibiting T cell senescence. Interactions between APCs and T cells determine the intensity of the immune response^139^. Therefore, restoring the function of APCs and T cells and their relationship is pivotal to improving the immune efficiency of elderly individuals. Determining how to simultaneously alleviate the immunosenescence of APCs and T cells to facilitate vaccine protection will be the focus.

Notably, natural killer (NK) cells are an important part of innate immunity and play an irreplaceable role in maintaining health, anti-infection, and antitumor functions in elderly individuals. Studies have shown that NK cells can recognize and clear senescent cells in a variety of ways, such as direct killing and the secretion of cytokines or perforins^140^. These findings imply that increasing the amount and improving the function of NK cells may be another option to promote the efficiency of vaccines based on anti-aging strategies.

### Current design strategies and examples

**Fig. 4:**
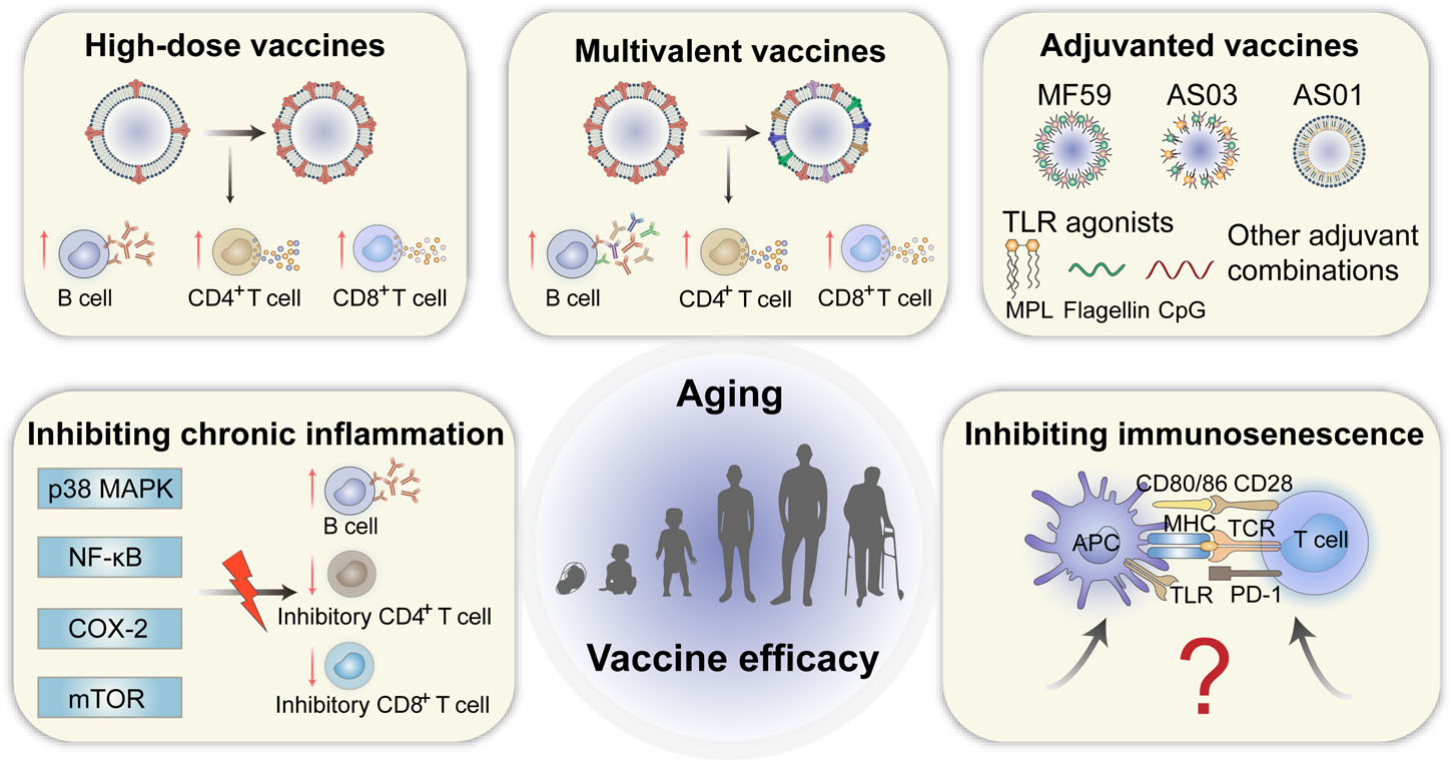
Vaccine design strategies for elderly individuals include increasing the dose of antigen, preparing multivalent antigen vaccines, adding appropriate adjuvants, inhibiting chronic inflammation, and inhibiting immunosenescence. AS adjuvant system, MPL monophosphoryl lipid, TLR toll-like receptor, MAPK mitogen-activated protein kinase, NF-κB nuclear factor kappa-light-chain-enhancer of activated B cells; COX cyclooxygenase; mTOR mammalian target of rapamycin; APC antigen-presenting cell; MHC major histocompatibility complex; TCR T cell receptor; PD-1 programmed death receptor-1. (Created with Adobe illustrator).

The current design strategies and examples of vaccines for elderly individuals are shown in Fig. 4 and Table 1. The differences in the effectiveness of the vaccine in older people also depend on whether it is a primary or booster vaccination. Primary vaccination with the herpes zoster subunit vaccine and the COVID-19 mRNA vaccine also resulted in high efficacy in the elderly population. Recombinant subunit herpes zoster vaccine can provide effective immune protection due to the adjuvant AS01, which is essential for improving immunity in the elderly. COVID-19 mRNA vaccines were approved for emergency use during the COVID-19 pandemic^141^. The mechanism may be that mRNA can be recognized by TLR3, 7, and 8 to induce adjuvant-like activity and enhanced antigen expression and antigen presentation^8^. Therefore, there is a strong interest in using mRNA vaccines against other diseases in the elderly. Moreover, booster vaccination may offset the decline in vaccine responses in older adults. When older adults receive two doses of BNT162b2 vaccine three weeks apart, the neutralizing antibodies of SARS-CoV-2 weaken over time, but a third booster dose of the vaccine can further increase antibody levels and cross-protection against Omicron BA.1 and BA.2^142^. Meanwhile, the interval between the two doses may also influence the effectiveness of the vaccines. In a population-based cohort study, participants were boosted with the BNT162b2 vaccine at three weeks or longer intervals (11–12 weeks). The results showed that the peak antibody response was 3.5-fold higher in participants who received the delayed interval vaccination^143^.

**Table 1:** Examples of vaccine design strategies for older adults

Design strategy	Pathogen	Vaccine information	References
High-dose vaccines	Influenza virus	Fluzone ® (provide four times more antigen)	^79^
Multivalent vaccines	*Streptococcus pneumoniae*	PCV13 (13-valent pneumococcal conjugate vaccine)	^90^
	*Streptococcus pneumoniae*	PPSV23 (23-valent pneumococcal polysaccharide vaccine)	^91^
Adjuvanted vaccines	Influenza virus	Fluad ® (containing MF59 adjuvant)	^97^ ^98^ ^99^
	Influenza virus	Pandemrix® (containing AS03 adjuvant)	^100^ ^101^
	Influenza virus	Containing Matrix M-1 saponin complex adjuvant	NCT03293498
	Influenza virus	Containing TLR 7/8 agonist resiquimod	NCT01737580
	Herpes zoster	Shingrix ® (containing AS01 adjuvant)	^104^
	Respiratory syncytial virus	Arexvy ® (containing AS01 adjuvant)	https://www.fda.gov/news-events/press-announcements/fda-approves-first-respiratory-syncytial-virus-rsv-vaccine
	SARS-CoV-2	TLR9 agonists synergizes with aluminum hydroxide adjuvant	^114^
Inhibiting chronic inflammation	/	p38 MAPK inhibitor	^115^
	/	mTOR inhibitor (rapamycin)	NCT02874924
	/	COX-2 inhibitor	^125^
	/	NF-κB inhibitor	^130^
Inhibiting immunosenescence	SARS-CoV-2 or influenza virus	Spermidine (Dietary Supplement) to restore autophagy in T and B cells	NCT05421546
	Influenza virus	Metformin to restore autophagy in T and B cells	NCT03996538
	*Streptococcus pneumoniae*	Metformin to restore autophagy in T and B cells	NCT03713801
mRNA vaccines	SARS-CoV-2	BNT162b2 and mRNA-1273	^141^
Boost vaccination	SARS-CoV-2	A third booster dose	^142^
Delayed interval vaccination	SARS-CoV-2	The interval between the two doses was extended from 3 weeks to 11-12 weeks	^143^
Intradermal injection	Influenza virus	Intanza ® (increase antigen presentation)	^146^

*AS* adjuvant system, *TLR* toll-like receptor, *MAPK* mitogen-activated protein kinase, *NF-κB* nuclear factor kappa-light-chain-enhancer of activated B cells, *COX* cyclooxygenase, *mTOR* mammalian target of rapamycin.

## Conclusions and perspectives

The global population is entering an era of aging. Older people are more susceptible to pathogens and have higher rates of morbidity and mortality^144^. Despite the significant success of current vaccine products, many commercial vaccines fail to generate effective and long-lasting immune protection in elderly individuals. With increasing age, the reasons for the decline in vaccine potency are multifactorial. Age-related dysregulation of lymph nodes, and crucial immune cells jointly reduces the efficiency of vaccination. With the continuous emergence of new pathogens, it is urgent to create strategies to improve vaccination-mediated protection for elderly individuals.

This review summarized a series of approaches to provide a reference for vaccine design for elderly individuals. We first analyzed the characteristics of immunosenescence in the elderly population and outlined the impacts of these changes on the potency of vaccination. The main measures to enhance vaccination efficiency in the elderly are increasing the antigen dose, preparing multivalent vaccines, multiple immunizations, and adding adjuvants to enhance immune responses. Subsequently, we discussed current strategies for improving immune responses to vaccines, analyzed their advantages and limitations, and proposed potential directions for future research.

The existing approaches are primarily aimed at optimizing the vaccine delivery system rather than inhibiting the immunosenescence of the immune microenvironment in elderly individuals. Inhibiting the immunosenescence of elderly individuals can evoke strong and long-lasting immune protection, which serves as a critical measure to improve vaccine-induced immunity. Although inhibition of immunosenescence most likely requires continuous intervention/treatment and is complicated to achieve, we believe that sustained-release vaccination/adjuvants or booster immunizations may sustainably ameliorate immunosenescence in the elderly. Once the immunosenescence of the elderly is corrected, their immune efficacy against various antigens can be improved. An attractive research direction will be discovering immunomodulators and vaccine formulations that can inhibit immunosenescence. The selection of adjuvants can greatly impact the type and magnitude of the immune response^145^. Considering the special immune status of elderly individuals, designing tailored vaccine adjuvants is indispensable for the development of next-generation vaccines for older individuals. A chronic inflammatory state also accompanies immunosenescence. However, the common opinion is that adjuvants promote immunity by inducing local inflammation. Therefore, more in-depth studies are needed to explain the role of inflammation in vaccine-induced immunity and tune the contradictory perspectives.

In addition to the described methods to improve vaccination efficiency, there are also studies to alter the route of administration. Vaccines are generally injected subcutaneously and intramuscularly. Some studies have explored the efficacy of vaccines administered via other routes. Clinical studies have indicated that intradermally injecting influenza vaccine can improve the titer and seroconversion rates in elderly individuals after vaccination^146^. The dermis contains many specialized DCs, such as LCs, which can present antigens^26^. However, an intradermal injection can cause more pain and local adverse reactions. The mucosal routes, including oral, intranasal, and vaginal vaccination, may be promising approaches. A study comparing intranasal and intramuscular vaccination in older populations showed that seroconversion rates are similar, but intranasal vaccination can generate more mucosal IgA responses^147^. For efficient nasal delivery, vaccines should be internalized by microfold cells and DCs in nasal-associated lymphoid tissues^148^. In addition, vaccine design needs to account for more factors, such as the limited volume of administration and the movement of nasal cilia leading to vaccine clearance. Thus, it is necessary to study alternative vaccination routes for elderly individuals further.

During clinical research, observing different immune outcomes in animal models and human settings is common. The characteristics and functional differences in anatomical structures, disease models, and TLR expression profiles between humans and mice have pivotal impacts. Additionally, many aging-related experiments in elderly mice were performed under specific pathogen-free conditions, which have some limitations. Older individuals may be in a frail state with multiple underlying diseases and comorbidities, which may also affect vaccine potency^18,149^. The ongoing evolution and immune imprinting of influenza viruses and SARS-CoV-2 strains is an important impediment to the effectiveness of vaccines against new variants^150^. Once the immune system has built up an immune memory for an antigen, when it encounters a new, similar antigen, it will preferentially activate the immune response against the previous antigen. It is desirable if a response to a shared epitope results in virus neutralization, but it can be detrimental if a response to a non-protective epitope is dominant. In aged mice, immune imprinting of influenza virus reduced the efficacy of the vaccine against *Streptococcus pneumoniae*^151^. Wang et al. developed a method that measures the antigenic distance between different strains to predict the immune response to a particular strain^152^. Animal studies suggest that using dendritic cell-activating adjuvants and repeated immunization may overcome immune imprinting^153^, and these measures can also improve the efficacy of the vaccine in the elderly. We believe that the ideal vaccine response would overcome immune imprinting and combat a broader spectrum of pathogens and variants. Such universal vaccines could prevent the virus from mutating to the point where it escapes the immune system and could ultimately be the key to controlling future pandemics.

Most studies focus on one or several cell types or certain processes of the immune response. However, our immune system is a complex and coordinated comprehensive network. More new technologies and advances will help reveal the complexity underlying the human immune system. We must pay more attention to the impacts of versatile cells or multiple immune cascade processes. Future research should focus on developing scientific methods to build more convincing models of aging and study the profound mechanisms underlying age-related alterations that impact the immune responses of older people. Additionally, interdisciplinary research involving vaccinology, immunology, and artificial intelligence may also assist in the research and development of vaccines; this collaboration could provide more innovative strategies and spark ideas for personalized vaccine design.

## Data Availability

Data availability is not applicable to this article as no new data were created or analyzed in this study.
